# Immunological Causes of Infertility: Diagnostic Perspectives

**DOI:** 10.3390/biom16010039

**Published:** 2025-12-25

**Authors:** Aleksandra M. Kicińska, Radosław B. Maksym, Grzegorz Szewczyk

**Affiliations:** 1 Medical Faculty, Medical University of Gdańsk, 80-210 Gdańsk, Poland; 2 1st Department of Obstetrics and Gynecology, Centre of Postgraduate Medical Education, 01-004 Warsaw, Poland; radoslaw.maksym@cmkp.edu.pl

**Keywords:** infertility, reproductive immunology, autoimmune diseases, t-lymphocytes, progesterone, endometriosis, chronic endometritis, cytokines, natural killer cells, antisperm antibodies, inflammation

## Abstract

From an immunological perspective, infertility mechanisms encompass not only fertilization but also implantation, as well as both early and late pregnancy loss. Growing attention is being directed towards the influence of systemic disorders on reproductive outcomes. The immune system plays a fundamental and regulatory role in human reproduction. Immunological factors may affect multiple stages of this process, potentially justifying their inclusion in extended diagnostic pathways. The impact of autoimmunity and the presence of various antibodies on reproductive functions is discussed. Special attention is given to the immunomodulatory role of progesterone in reproduction and a state of impaired progesterone action—luteal deficiency. Endometriosis is also highlighted as a disorder both associated with infertility and underpinned by a strong immunological basis. The usefulness of assessing lymphocyte subpopulation balance, cytokine profiles, and Th1/Th2 immune response in the diagnostic work-up of infertility is addressed. Furthermore, the prospect for a role of local and systemic infections, subclinical inflammation and microbial colonization is shown. Tests applied in the evaluation of implantation and placental development disorders are discussed. Adequate immunological diagnostics and accurate identification of the underlying causes of infertility facilitate effective therapeutic strategies and can substantially increase the likelihood of achieving a successful pregnancy.

## 1. Introduction

Infertility represents an ever-growing problem worldwide. Recognized by the World Health Organization as a lifestyle-related disease, it is defined as the inability to achieve pregnancy within one year of regular intercourse (2–4 times per week) without the use of contraception. From an immunological perspective, the problem encompasses not only fertilization but also implantation, as well as both early and late pregnancy loss. Nearly one in five couples experience difficulties in conceiving. Immunological diagnostics is not part of routine first-line infertility assessment but may be a significant extension of in-depth diagnostics for selected couples. Evaluation encompasses all stages of reproduction: ovulatory disorders, impaired spermatogenesis, recurrent implantation failure (RIF), recurrent pregnancy loss (RPL), disturbances in fetal growth, complications within the spectrum of preeclampsia, and preterm birth. Inadequate tolerance or excessive activity of immunocompetent cells at any of these stages may lead to immunological infertility or obstetric complications, conditions that are frequently classified as idiopathic infertility or disorders of unknown etiology. For this reason, novel diagnostic approaches are increasingly sought to enable effective treatment for infertile couples. Reproductive medicine is increasingly turning to diagnostic tools for the immune system and its disorders that interfere with reproduction. However, their interpretation is often challenging, results can be ambiguous, and their isolated use without consideration of systemic medicine may lead to incorrect conclusions.

Accordingly, this review aims to summarize key immunological mechanisms underlying reproductive processes and to outline systemic disorders that exert the greatest impact on the immunological basis of reproduction. Beginning with physiological fundamentals, we reviewed recent literature to better understand immunometabolic and hormonal interactions, as well as the impact of systemic diseases and infections on fertility disorders. The literature search using PubMed^®^ (http://pubmed.ncbi.nlm.nih.gov/ (accessed on 1 November 2025)) was guided by different combinations of the following keywords: infertility; reproductive immunology; autoimmune diseases; T lymphocytes; progesterone; endometriosis; chronic endometritis; cytokines; natural killer cells; antisperm antibodies; inflammation.

We describe key reproductive processes: ovulation, implantation, and the endometrial response to sperm exposure, to provide foundational insight into the immune factors essential for clinical reasoning and the implementation of appropriate diagnostic and therapeutic pathways.

Diagnostic evaluation of immunological infertility should begin with the exclusion of systemic and local inflammatory processes within the reproductive tract, including chronic endometritis, autoimmune diseases, nutritional deficiencies, and disturbances in estrogen-progesterone homeostasis. Ongoing debate exists regarding the clinical value and positioning of tests assessing lymphocyte subpopulations and cytotoxicity, and maternal immune responses to paternal and embryonic antigens, owing to their methodological and interpretative limitations.

**Figure 1:**
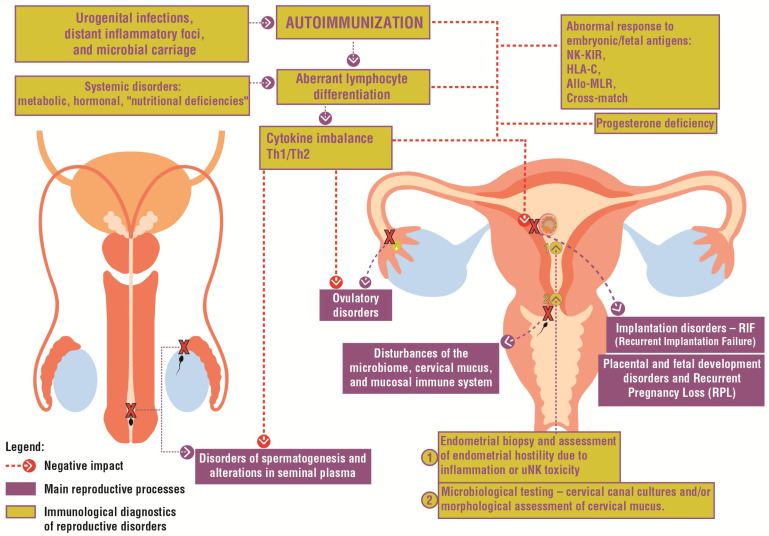
Immunological diagnostics of infertility. The mucous membranes of the reproductive tract, especially the vaginal and cervical epithelium, are characterized by disturbances in locally secreted immunological factors as well as increased sensitivity and susceptibility to the permeability of microorganisms and their toxins, particularly in the presence of hormonal disorders and bacterial dysbiosis [3]. Any type of carriage and colonization by pathogenic viruses and bacteria may contribute to alterations in the mucus secreted by the cervical crypts. This disrupts the formation of a suitable environment favorable for sperm, allowing them to reach the oocyte in the ampulla of the fallopian tube and fertilize it. The presence of pathogens, however, generates a local defensive immune response through the influx of cytokines and inflammatory mediators into the cervical crypts and mucus secretions [4,5]. Clinically, this may manifest as discomfort, discharge, or lower abdominal pain, or it may remain asymptomatic, yet still contribute to infertility [6]. The immunological properties of cervical mucus have a profound effect on fertility. If the balance dependent on the proper hormonal course of the cycle is disrupted, infertility may arise at the level of immunological disturbances within the cervical mucus. Various antibodies may appear, such as antisperm antibodies (ASA) or antibodies against the oocyte’s zona pellucida, which hinder reproductive processes. In women with unexplained infertility, antisperm antibody activity has been detected in cervical mucus in more than 10% of cases [7]. Antisperm antibodies disrupt the interaction of sperm with the mucus, preventing the proper course of capacitation; they induce cytokine production and cause agglutination of sperm in the mucus. ASA can identify specific antigens on sperm, impairing their function, including motility, capacitation, acrosome reaction, and penetration into the oocyte. Other antibodies, not specific to gamete structures, have also been detected in the mucus of infertile women, yet they significantly interfere with reproductive processes. These include, for example, antibodies against Helicobacter pylori [8]. A correlation has been shown between the presence of IgG antibodies against Helicobacter pylori (anti-H. pylori) in the serum and cervical mucus of women with idiopathic infertility. The impact of these antibodies on cervical mucus quality has also been demonstrated, particularly in relation to the reduced ability of sperm to penetrate.

**Figure 2:**
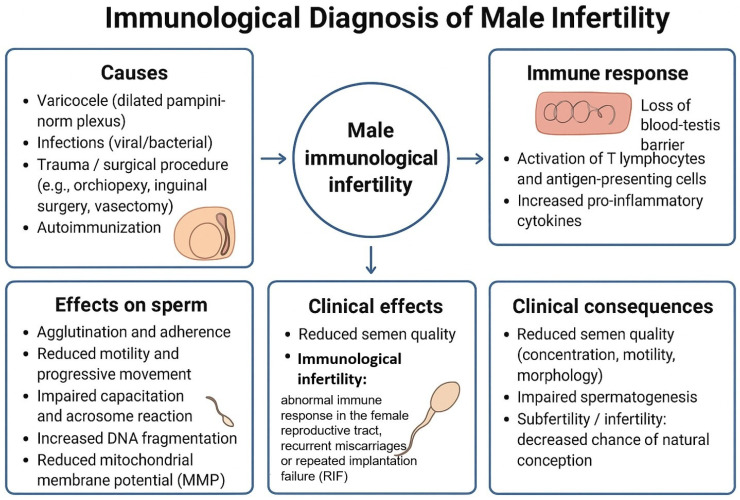
Immunological diagnosis of male infertility. The main causes and immunological mechanisms involved in male infertility are listed in the figure.

The general health status of both women and men must also be considered, particularly the influence of systemic diseases on immune function and fertility (Figure 1). Among the most common causes of female infertility with a significant immunological component are endometriosis and polycystic ovary syndrome. Issues relating to immunological disturbances and therapeutic possibilities in these conditions have been thoroughly and extensively discussed in recent publications [1,2]. In the present review, we aim to complement existing knowledge on immunological determinants of infertility, with particular attention to male factors (Figure 2).

## 2. Immunological Aspects of the Menstrual Cycle and Reproductive Processes

The immune system plays a fundamental and regulatory role in processes throughout the body, including those within the reproductive system. Key processes essential for fertility—spermatogenesis, oogenesis, ovulation, endometrial regeneration, fertilization, implantation, and placental development—are associated with the occurrence of a controlled inflammatory state regulated by immune cells. Appropriate modulation of the immune response, dependent on the overall systemic condition of the organism, ensures the resolution of local inflammation and the success of reproduction. Immune, endocrine, and nervous system cells share common ligands and receptors, and the close interactions between them form the neuro–immune–endocrine network, which supervises the function of reproductive processes [9].

The culmination of female fertility is the development of a competent oocyte and its release during ovulation. Folliculogenesis within the ovarian tissue, combined with appropriate signal transduction between immune cells, ensures the release of a mature and functionally competent oocyte from the dominant follicle. Recruitment and influx of leukocytes into the follicular wall initiate extracellular matrix remodeling and the rupture of the Graafian follicle, tunica albuginea, and ovarian surface epithelium. The processes occurring in the ovary currently possess a distinct inflammatory component. Peripheral leukopenia, neutrophil dysfunction, and other immunological disturbances may impair this process. A typical immunologically mediated ovulatory disorder is the luteinized unruptured follicle (LUF) syndrome, most associated with endometriosis [2]. In contrast, appropriate luteinizing hormone (LH) signaling stimulates granulosa cells to secrete chemokines locally, which in turn initiate the release of regulatory molecules necessary for resolving inflammation. This protects the oocyte from the destructive effects of pro-inflammatory cytokines, thereby supporting successful fertilization and reproduction [10].

The stages of fertilization, embryo transport, and implantation are also under strict immunological control. From the earliest stages of its development, the embryo secretes chorionic gonadotropin (hCG) and the pro-inflammatory cytokine interleukin-1-beta isoform (IL-1β), which induce transformation of the endometrial epithelium and stimulate the corpus luteum to increase progesterone production, acting on receptors at the endometrial surface. This process triggers vascularization and changes in the transcriptional expression of cytokines, growth factors, chemokines, and adhesion molecules within the uterine decidua [11]. Immune cells from the maternal peripheral blood infiltrate the endometrium. Initially, implantation is dominated by Th1 lymphocytes, which promote a pro-inflammatory environment favoring embryonic invasion and penetration of syncytiotrophoblast cells into the decidua. However, precise resolution of inflammation is essential to prevent its excessive amplification and dissemination, which would damage the embryo and impair implantation. For this reason, maternal blocking antibodies and regulatory T cells (Tregs) are necessary to suppress Th1 activity. The essence of these processes determining reproductive success is immunomodulation—that is, a dynamic interaction between pro-inflammatory and anti-inflammatory responses—rather than the sequential action of Th1 and Th2 cells, as previously described in the Th1/Th2 paradigm [12].

Successful implantation depends on the actively developing balance between pro-inflammatory Th1 and Th17 cells and anti-inflammatory Th2/Treg cells, which in turn is modulated by progesterone. On the one hand, suppression of excessive activity of pro-inflammatory effector T cells (Th1 and Th17) by Tregs immediately after implantation is indispensable for further normal placental and pregnancy development. On the other hand, the increased activity of pro-inflammatory Th17 cells is a necessary factor in protecting the embryo and fetus. Maintenance of the correct Treg/Th17 ratio is critical for sustaining pregnancy, supporting its proper development, and preventing complications such as miscarriage, preeclampsia, and preterm birth [13].

Regulatory T cells (Tregs), a subset of CD4(+) suppressor T cells, play a dominant role in maintaining immunological tolerance by inhibiting both immune and autoimmune responses. Tregs are crucial for the establishment and maintenance of pregnancy, as they protect the semi-allogeneic fetus from rejection by the maternal immune system owing to the presence of paternal antigens. Unexplained infertility and pathological conditions during pregnancy are often associated with a deficiency in the number or function of Tregs [14].

An appropriate increase in Treg number and activity during pregnancy is dependent on multiple factors. Among the principal factors are maternal hormones, but also paternal seminal fluid, as well as the metabolic status and overall systemic condition of the mother.

In women with metabolic disturbances associated with hyperglycaemia and insulin resistance, increased glucose availability initiates the glycolytic pathway in T lymphocytes, promoting their differentiation towards a Th1/Th17 phenotype while suppressing the generation of regulatory T cells (Tregs). Alterations in Treg number and function may also be influenced by the presence of pathogens and an abnormal reproductive tract microbiome, vitamin D3 deficiency and its lost anti-inflammatory effects, as well as excessive intake of simple sugars in the diet.

Overnutrition, obesity, and systemic inflammatory states of various origins lead to mitochondrial dysfunction and excessive production of reactive oxygen species (ROS). Therefore, elevated mitochondrial oxidative stress induces a state of chronic low-grade systemic inflammation, disrupting both the number and functional activity of Treg lymphocytes. This results in heightened immune responses, an increased risk of autoimmune diseases, and dysregulation of immunological factors crucial for proper implantation, placental development, and embryonic and fetal growth. Low-grade inflammation also extends to ovarian tissue, creating a hostile environment for folliculogenesis, thereby reducing oocyte quality.

The multifaceted mechanisms of immunoregulation in reproductive processes arise from the interplay between local microenvironment within the ovary and endometrium and systemic factors that shape fertility potential. Immunometabolic relationships throughout the organism influence the production and action of hormones, cytokines, and adipokines, which collectively determine reproductive success [1]. Within these processes, Tregs perform a principal regulatory role.

Treg lymphocytes interact with multiple innate immune cells present in the uterine mucosa at the time of implantation, particularly macrophages, dendritic cells, and unique populations of uterine natural killer (uNK) cells with the CD56+ phenotype. These cells acquire anti-inflammatory phenotypes in response to modulation by Tregs, in conjunction with progesterone and unique signals derived from the trophoblast [7,15].

Rising concentrations of estrogens and progesterone during pregnancy contribute to a shift in the immune response from a predominantly pro-inflammatory Th1 and Th17 immune profile towards a Th2/Treg profile, anti-inflammatory phenotype. Both Th1 and Th17 lymphocytes are pro-inflammatory, although they exert distinct biological functions. Th1 lymphocytes contribute to transplant rejection through the production of IL-2, tumor necrosis factor-alpha (TNF-α), and interferon-gamma (IFN-γ) and participate in the regulation of trophoblast invasion as well as tissue remodeling and angiogenesis during implantation. In contrast, Th17 cells are characterized by the production of interleukin-17 (IL-17) and contribute to host defense against pathogens as the material-fetal interface during early implantation [16].

Progesterone, through binding to its receptor within the endometrium, suppresses lymphocyte cytotoxicity and exerts anti-inflammatory effects [17,18]. Lymphocytes expressing specific progesterone receptors begin producing the progesterone-induced blocking factor (PIBF), which enhances the production of Th2 cytokines while inhibiting uNK degranulation, arachidonic acid release, and the synthesis of prostaglandins and leukotrienes [19]. At this stage, uterine natural killer (uNK) cells play a pivotal role, together with dendritic cells (DCs), in initiating the transformation of spiral arteries for placental development and in supervising the establishment of an appropriate cytokine profile at the maternal–fetal interface. Under the influence of progesterone, uNK cells and DCs induce the differentiation of naïve T lymphocytes into regulatory T cells (Tregs), which are critical for successful implantation and the development of a normal placenta. Deficiency of the immunomodulatory effects of progesterone leads to disturbances in cytokine balance, which may ultimately result in recurrent implantation failure (RIF) as well as abnormal placental, pregnancy, and fetal development [20]. Progesterone prevents uNK cytotoxicity and promotes the generation of Tregs, thereby protecting the embryo from immune rejection. Therefore, the evaluation or assessment of progesterone activity to determine the causes of immunological infertility should be considered in all cases of reproductive failure, to allow for adequate supplementation of this immunomodulatory hormone. Importantly, in infertile women attempting conception, progesterone supplementation should be initiated after ovulation rather than from a rigidly predetermined day of the cycle. As it was previously demonstrated, personalization of the timing of progesterone administration may be crucial to therapeutic success [4].

Male infertility of immunological origin may involve impaired spermatogenesis, defective sperm maturation, subsequent sperm damage, and abnormalities in seminal plasma composition. Sperm production takes place in the testes, which—being located outside the blood–testis barrier—constitute an immunologically privileged organ. Pathological conditions such as trauma, infections, varicocele, or other defects that disrupt the blood–testis barrier may trigger immune sensitization or promote infection, leading to reduced sperm quantity and quality in the ejaculate and the development of immunological infertility (Figure 2). Similarly, infections may affect the accessory glands of the male genital tract (male accessory gland infections, MAGI) [21]. In addition, systemic disorders such as metabolic syndrome, nutritional, vitamin, and micronutrient deficiencies, and other conditions that provoke low-grade inflammation contribute to oxidative stress, resulting in decreased ejaculate quality [22].

Following intercourse, seminal fluid not only acts as a carrier ensuring sperm survival and fertilization capacity, but also modulates the female reproductive system, enhancing the likelihood of conception, implantation, and pregnancy maintenance. Insufficient exposure to the ejaculate of the prospective father may impair the signaling and immunological priming of the female reproductive tract required for the acceptance of the embryo, which constitutes a semi-allogeneic graft containing paternal antigens—the same antigens present in semen. During exposure of the genital mucosa to semen, an essential “immunological memory” is established, programming tolerance towards partner-derived antigens, a process that is reinforced with each subsequent intercourse [23]. Seminal plasma introduces into the female organism a wide range of immunologically active factors, including cytokines and prostaglandins synthesized in the male accessory glands, which, by interacting with receptors in the genital mucosa, induce changes in gene expression. Seminal plasma plays a critical role in recruiting and expanding the pool of leukocytes, including regulatory T cells, within the uterus prior to implantation. Notably, seminal plasma contains soluble CD38 (sCD38), produced in the seminal vesicles, which acts in the female genital tract as a potent inducer of tolerogenic dendritic cells and CD4+Foxp3+ Tregs. An important soluble factor in semen that promotes embryo tolerance mechanisms within the female reproductive tract is HLA-G, produced by the testes and epididymis [24,25]. The processes described above lead to the activation of adaptive mechanisms through the establishment of immunological tolerance, thereby facilitating implantation and embryonic development despite the presence of paternal antigens. A similar interaction also occurs directly between spermatozoa and cells of the female reproductive tract [26]. Excessively prolonged sexual abstinence may be unfavorable, not only because of reduced sperm quality parameters, but also due to an inadequate maternal immune response to sperm and embryonic antigens.

Recognition and investigation of the impact of those immunological factors on disturbances at different stages of reproduction may constitute a consistent component of the diagnostic evaluation of infertile couples.

## 3. Autoimmune Diseases and Autoimmunization (Presence of Autoantibodies)

Autoimmune diseases exert a negative impact on fertility and pregnancy outcomes. They occur more frequently in women of reproductive age, which is associated with the activating effects of estrogens on immune cells. Baseline, low concentrations of estrogens stimulate the production of pro-inflammatory cytokines, whereas high peri-ovulatory concentrations of estradiol promote the production of anti-inflammatory cytokines and enhance the Th2-dependent humoral immune response. With rising estrogens levels, the activity of cytotoxic T lymphocytes decreases markedly, while the proportion of regulatory T cells (Tregs) increases, ensuring the correct course of ovulation [27]. In the second half of the cycle, when the corpus luteum produces progesterone, the expression of membrane receptors for this hormone on cytotoxic lymphocytes (CD8+) increases, contributing to the suppression of cytotoxic immune responses by modulating the Th1/Th2 balance [17]. Both estradiol and progesterone exert effects on lymphocyte function locally and systemically, thereby modulating immune responses throughout the body [18].

Proper hormonal fluctuation during the cycle is the first prerequisite for creating an immunological environment favorable to reproduction. Disturbances in the balance between estrogen and progesterone lead to a disruption between effector and regulatory immune responses, thereby providing the basis for autoimmunization [9]. Among infertile women, polycystic ovary syndrome (PCOS) and endometriosis frequently coexist, conditions associated with disruption of the estrogen–progesterone balance, which consequently initiates the production of autoantibodies [1]. At this stage, the presence of autoantibodies does not always equate to clinically manifest autoimmune disease; however, their mere presence is sufficient to impair reproductive success. Deficiency of the immunomodulatory effects of progesterone in women with irregular cycles or luteal phase defects increases the risk of autoimmunization, autoimmune diseases, and the potential development of malignancies [19]. Progesterone induces both local and systemic immunological tolerance through its action on glucocorticoid receptors expressed on Tregs [18].

The propensity for autoimmunization generally increases with age, arising from multiple factors: microbial carriage and chronic infections, persistence and amplification of immunological memory, and long-term exposure to immunogenic substances, including endocrine disruptors. Prolonged exposure to stressors activates the immune system, raising concentrations of pro-inflammatory mediators and leading to the accumulation of reactive oxygen species (ROS), pathological protein aggregation, and damage to nuclear and mitochondrial genetic material, including genetic mutations. This results in progressive mitochondrial dysfunction and telomere shortening due to insufficient DNA protection. A state of chronic low-grade inflammation ensues, leading to so-called “inflammaging” Consequently, aging affects the organism as a whole, manifesting as a decline in cellular function and structural integrity, including gametes, ultimately reducing reproductive potential [28]. It is noteworthy in this context that the greatest risk factor for infertility is advanced maternal age, followed by other determinants [29].

In autoimmunization, including that acquired with age, autoreactive B- and T-cell clones are induced, resulting in persistent autoantibody production. These antibodies trigger autoimmune processes that culminate in clinical syndromes. Such diseases include connective tissue disorders such as rheumatoid arthritis, Sjögren’s syndrome, systemic lupus erythematosus, autoimmune thyroiditis, diabetes mellitus, coeliac disease, antiphospholipid syndrome, and systemic vasculitis. In these conditions, both the presence of autoantibodies and the local and systemic abnormalities they provoke disrupt reproductive processes [30]. Autoantibodies act destructively at the cellular level, inducing overproduction of free radicals and increased oxidative potential. This leads to ovarian tissue inflammation and activation of pathomechanisms causing impaired follicular growth and rupture, diminished ovarian reserve and, ultimately, infertility [28].

A variety of autoantibodies (e.g., antinuclear, anti-FSH, or anti-hCG) are frequently present in apparently clinically healthy women who experience recurrent pregnancy loss, endometriosis, premature ovarian insufficiency (POI), unexplained infertility, or repeated failure of in vitro fertilization (IVF) embryo transfers [31]. Asymptomatic autoantibody presence, as well as clinically manifest autoimmune processes occurring in various tissues, may contribute to lymphocytic oophoritis and the occurrence of ovarian and endometrial autoantibodies. A strong association exists between autoimmunization and premature ovarian insufficiency (POI) [32]. Similarly, iron overload resulting from supplementation or from hypomenorrhoea increases exposure to free radicals generated through the Fenton reaction, and may be linked to earlier menopause and the development of cardiovascular disease [33,34].

Thyroid dysfunction and microcirculatory disturbances are particularly often detected among women with idiopathic infertility. Therefore, the presence of anti-thyroid antibodies—including anti-thyroid peroxidase (anti-TPO), anti-thyroglobulin (anti-TG), and anti-thyrotropin receptor antibodies (TRAbs)—as well as antibodies against endothelial membranes (anti-β2-glycoprotein, antiphosphatidylserine, annexin V, anticardiolipin, and circulating anticoagulant) may be of considerable interest for future research [20,35]. Systemic vasculitis associated with autoantibodies, and, in particular, primary and secondary antiphospholipid syndrome (APS), exert a direct impact on the development of granulosa cells within the growing follicle, as well as on ovulation and implantation. Circulating autoimmune antibodies may disrupt microcirculation in the smallest vessels during embryonic invasion into the endometrium, thereby leading to implantation failure and abnormal placental formation [35]. The consequences may include preeclampsia and abnormal fetal development with intrauterine growth restriction, progressing to intravascular coagulation and embryonic or fetal demise. Moreover, APS itself constitutes a life- and health-threatening condition for the mother at any stage of pregnancy, delivery, or the puerperium [36].

In the diagnostic work-up of infertility, systemic diseases arising from immune system dysfunction should be considered. To this end, serological testing for specific antibodies associated with coeliac disease or Addison–Biermer disease may be warranted, and studies are ongoing concerning the use of tests for food hypersensitivities and allergies [37,38]. Such disorders may lead to impaired absorption and utilization of nutrients, vitamins, and micronutrients, chronic inflammation within the intestines and other organs, metabolic and hormonal disturbances, and intestinal dysbiosis, thereby disrupting reproductive processes at multiple stages [39,40]. These conditions may sometimes present with few or nonspecific symptoms, and patients may not consult appropriate specialists, remaining undiagnosed. Their long-term clinical consequences are often first reflected in impaired fertility potential, with the diagnosis only being made during in-depth investigations of infertility causes.

Contrary to previous assumptions, testing for anti-nuclear antibodies (ANA) is of considerable significance in infertility diagnostics. These specific gamma globulins, directed against nuclear components (e.g., histones, DNA, cytoplasm), are well established in the diagnosis of systemic connective tissue diseases, but may also arise in inflammatory processes unrelated to autoimmunization. Regardless of the presence of systemic disease symptoms, ANA in the body negatively affect fertility. The presence of ANA disrupts oocyte and embryo development, impairs fertilization, implantation, and placental development. ANA may also represent a potential cause of miscarriage or preterm birth, as they activate the complement system, induce inflammatory infiltration, and promote immune complex deposition at the maternal–fetal interface, thereby impairing placental blood flow [40]. ANA can further induce activation of plasmacytoid dendritic cells and enhance production of pro-inflammatory cytokines. These cytokines stimulate humoral immune responses and drive further ANA production, creating a vicious cycle of low-grade chronic inflammation that can contribute to infertility even at low ANA titres [1]. A vicious cycle is thus established, initiating a state of low-grade chronic inflammation that may contribute to infertility even at low titres of ANA [1].

In infertility diagnostics, testing for autoantibodies—particularly those with a documented impact on reproduction—may be warranted, even in the absence of overt clinical symptoms. Establishing a diagnosis and implementing targeted therapy, dietary modification, and achieving remission of autoimmune disease can significantly improve reproductive outcomes. Diagnostic evaluation of hypersensitivity and allergies, together with the application of targeted pharmacological and dietary interventions as well as desensitization procedures, may enhance the functioning of immunological factors that influence fertility. ESHRE guidelines state that antinuclear antibody (ANA) testing may be considered for explanatory purposes, as some studies—including small prospective cohorts—suggest that ANA positivity is associated with a poorer prognosis for pregnancy maintenance. However, it has not been demonstrated that a positive ANA result identifies a subgroup of women with recurrent pregnancy loss (RPL) who benefit from immunotherapy. The American Society for Reproductive Medicine (ASRM) guidelines do not recommend routine ANA screening, as its clinical role remains insufficiently established. Therefore, further well-designed, controlled studies, ideally randomized trials, are needed.

## 4. Chronic Inflammation and Infections

In the diagnostic evaluation of infertility of immunological origin, it is essential to exclude inflammatory foci throughout the entire body, not only within the genitourinary tract. Inflammatory states arising from acute or chronic infections, subclinical or asymptomatic infections, or microbial carriage promote immunological dysregulation [6]. Depending on the patient’s clinical presentation and symptoms, diagnostics should be directed towards identifying the source of infection. The most common inflammatory foci to be considered include those originating in the teeth, periodontium, paranasal sinuses, stomach, parasitic diseases, and intestinal dysbiosis, as well as inflammatory conditions of the urinary tract, bacterial vaginal dysbiosis, chronic inflammation of the cervix and endometrium, and inflammatory processes within the male reproductive tract [7,8,21,39].

Local activation of the immune system within inflamed tissue triggers further signaling pathways that stimulate lymphocytes towards cytotoxic activity, production of inflammatory mediators, and antibody synthesis. Increased production of pro-inflammatory cytokines (interleukin-1 (IL-1), tumor necross factor-alpha (TNF-α), interleukin-6 (IL-6), chemokines) may lead to acute inflammation or evolve into chronic low-grade inflammation disseminating throughout the organism. This mechanism underlies the pathogenesis of many metabolic disorders, atherosclerosis, cardiovascular diseases, neurodegenerative conditions, and malignancies [15]. Both the presence of microorganisms and infections, as well as disturbances in lipid and glucose metabolism, insulin resistance, excessive intake of simple sugars, and visceral obesity, generate oxidative stress and hormonal dysfunction, thereby contributing to the development of endothelial inflammation. By continuity, this inflammation spreads to other tissues and organs, including glandular tissue, gonads, and the uterus. In the ovary, it results in abnormal follicular growth, impaired oocyte maturation and ovulation, and disruption of endometrial receptivity; in men, it adversely affects spermatogenesis and sperm maturation. Chronic low-grade inflammation is associated with increased activity of cytotoxic T lymphocytes and elevated production of pro-inflammatory cytokines, accompanied by a reduction and dysfunction of regulatory T cells (Tregs), which play a central role in controlling immunological processes [1].

Inflammatory conditions of the reproductive tract exert a particularly significant impact on reproductive processes. The presence of pathogenic microorganisms has a direct toxic effect on the tissues and structures of the reproductive system and additionally promotes the influx of inflammatory mediators and antibodies into the mucosa [3].

In the setting of inflammation, the secretions of the endometrium and fallopian tubes, cervical crypts, and vaginal epithelium become toxic to spermatozoa, the oocyte, and the embryo, thereby contributing to infertility of immunological origin [41,42]. Microbial carriage within the cervix favors the production of abnormal cervical mucus, weakens the protective barrier, and disrupts the reproductive tract microbiome. Conversely, the absence of a normal microbiota deprives the organism of anti-inflammatory substances. Dysbiosis of the female reproductive tract constitutes a risk factor for recurrent miscarriage [43]. Cervical mucus represents the main component of vaginal secretions and the first line of defense against pathogens. Its composition directly influences the success of insemination, fertilization, and embryo implantation. Depending on the hormonal phase of the cycle, cervical mucus contains different immunological factors, including immunoglobulins IgG and IgA, lymphocytes, macrophages, prostaglandins, complement components, cytokines, lysozyme, calprotectin, lactoferrin, β-defensin, and others [44]. Disturbances in the estrogen–progesterone balance, as observed in women with irregular cycles, after menopause, and in adolescents with immature cycle regulation, predispose to increased susceptibility to microbial invasion, as they generate an abnormal composition of reproductive tract secretions [45]. This facilitates the transmission of sexually transmitted infections and reduces reproductive potential [46,47]. It has been demonstrated that cervical mucus in infertile women contains higher levels of inflammatory cytokines, such as IFN-γ, TNF-α, IL-6, and IL-8 [45]. A pro-inflammatory environment within the mucosa of the reproductive tract contributes to reduced oocyte quality, impaired transport of sperm and embryos, implantation failure, and placental dysfunction. Therefore, infections of the genitourinary tract should be investigated in infertile couples. *Chlamydia trachomatis*, *Ureaplasma* spp., and *Mycoplasma hominis* are uropathogens commonly detected in patients with primary infertility [48]. Ureaplasma parvum and Ureaplasma urealyticum are highly prevalent. Their asymptomatic presence can be identified in as many as 40–80% of sexually active women. These microorganisms may be found in both the female and male genitourinary tracts—including the vaginal mucosa, cervix, urethra, endometrium, seminal fluid, spermatozoa surfaces, as well as in amniotic fluid and placenta. However, some scientific reports describe Ureaplasma species as commensals of the reproductive tract with no effect on fertility [49,50]. Other studies, however, indicate that even asymptomatic colonization by Ureaplasma urealyticum or Ureaplasma parvum in sexually active women is associated with elevated levels of the pro-inflammatory cytokines IL-6 and IL-1β in the mucosa of the reproductive tract, with IL-6 concentrations being even higher in Ureaplasma parvum carriers compared with those colonized by Ureaplasma urealyticum [51].

Both IL-6 and IL-1β disrupt the local immune response in the female reproductive tract. These cytokines perpetuate inflammation, promote angiogenesis, and support the survival of ectopic lesions in endometriosis. Moreover, in their presence, T lymphocytes exhibit altered immunological responses, contributing to chronic inflammation [52].

Elevated levels of pro-inflammatory cytokines thereby prevent adequate leukocyte recruitment and hinder the generation of sufficient numbers and activity of regulatory T cells (Tregs), which are crucial at multiple stages of reproduction—from the response to spermatozoa to embryo implantation and normal pregnancy progression. Increased IL-6 levels have been associated with preterm birth, cervical inflammation, and altered vaginal microbiota [53,54].

IL-1β is present intracellularly in epithelium at sites of cellular damage and plays a central role in regulating the inflammatory response in injured tissue. IL-1β also participates in the development of preterm labor induced by infection-driven excessive prostaglandin production in the amniotic epithelium [55].

Both IL-6 and IL-1β are key inflammatory mediators of coagulation. Consequently, they may contribute to implantation disorders by altering blood rheology in the developing syncytiotrophoblast and placenta [56].

Because reproductive processes are tightly regulated by inflammatory mechanisms, precise control over their initiation and resolution is essential. Exaggerated or premature activation of inflammation can contribute to reproductive failure. For this reason, detection and treatment of microorganisms that disturb cytokine balance appear to be important and necessary measures in reproductive medicine.

It is crucial that microbiological diagnostics be broad-spectrum, directed towards various pathogens, and employ culture on specialized media, PCR methods, and immunohistochemical techniques [7]. Vaginal and cervical canal microbiological testing can be readily performed clinically by swabbing, whereas endometrial assessment requires uterine lavage or endometrial biopsy. To some extent, the results of cervical swab and culture reflect, by continuity, the microbiological status of the endometrial cavity. Antibiotic treatment of chronic endometritis and reproductive tract infections facilitates proper sperm transport and capacitation and increases the likelihood of successful embryo implantation [7,57]. In situations where specific bacteria are present, bacterial load fluctuates, or antimicrobial therapy precedes culture sampling, the likelihood of false-negative results increases [57]. In many instances, clinical evaluation of reproductive tract secretions—comprising a mixture of secretions from the uterine cavity, cervix, and vagina—can more reliably indicate the presence of inflammatory conditions in the reproductive tract mucosa. Such evaluation should be performed according to a standardized method of cycle observation to avoid the risk of misinterpretation [7]. In justified cases, this may indicate the need for antibiotic therapy to restore a normal microbiome and immunological balance in infertile couples [57].

Attention should also be given to infections caused by sexually transmitted bacteria or uropathogens, which induce epididymitis and orchitis and constitute a major factor contributing to male infertility. On the one hand, these conditions impair sperm quality and quantity; on the other, they modify the composition of seminal plasma entering the female reproductive tract. Infected semen may act as a source of microbial transmission, with obvious consequences for female reproductive tract fertility, as described above. Furthermore, through its increased content of bacterial toxins and pro-inflammatory cytokines, infected semen may impair the mechanisms responsible for establishing immunological tolerance to the embryo within the female reproductive tract. Therefore, it is essential to investigate the complex mechanisms underlying the pathogenesis of infections and inflammation in the male reproductive tract, which generate elevated levels of reactive oxygen species (ROS) [58]. Not only inflammation of the accessory male glands, testes, and epididymis, but also vasectomy, testicular trauma, and previous surgical interventions may trigger inflammatory responses leading to the production of anti-sperm antibodies (ASA) and oxidative stress. Both ROS and sperm-immobilizing antibodies significantly reduce fertility potential by inhibiting sperm motility and migration in the female reproductive tract, ultimately impairing reproductive outcomes for the couple [59].

Many immunological disorders and potential therapeutic approaches are associated with chronic disease with a strong immunological component—namely endometriosis. The immune system is involved both in the development of endometriosis and in the mechanisms by which it affects fertility. Some of the best-documented methods of improving fertility through immune-modulating interventions—such as hysterosalpingography with oil-based contrast medium or surgical excision of endometriotic lesions—specifically concern endometriosis. The complexity of immunological mechanisms related to endometriosis and the evidence for the efficacy of therapeutic approaches have been described previously [2].

However, observational studies have demonstrated an increased incidence of chronic endometritis among women with endometriosis [60]. Chronic endometritis and endometriosis are distinct yet related chronic inflammatory conditions of uterine tissue. Both may cause pelvic pain and infertility, share overlapping immune-mediated mechanisms, and increase the risk of implantation failure and miscarriage.

Chronic endometritis, unlike endometriosis, represents inflammation of the lining of the uterine mucosa characterized by infiltration of CD138-positive plasma cells and is most commonly associated with infection or dysbiosis. Endometriosis, in contrast, is defined by the presence of ectopic endometrial-like tissue outside the uterine cavity, including peritoneal and ovarian lesions. Deep infiltrating endometriosis constitutes a distinct clinical entity, penetrating tissues beneath the peritoneum and potentially impairing the function of the intestines, ureters, bladder, nerves, uterosacral ligaments, and rectovaginal septum.

Adenomyosis, characterized by the infiltration of endometrial glands into the myometrium, is closely related to endometriosis. Adenomyosis frequently coexists with various forms of endometriosis, and their etiopathogenesis is likely shared. Both endometriosis and adenomyosis initiate extensive inflammatory responses in the pelvis and in reproductive tract lumen [2].

Endometriosis significantly increases the risk of coexisting chronic endometritis, supporting the concept of shared immune signaling pathways and inflammatory mechanisms [61]. Consequently, the need for integrated diagnostic and therapeutic strategies addressing both conditions should not be overlooked. Such an approach may yield measurable clinical benefits for patients presenting with infertility or menstrual cycle disorders. Understanding the immunological and microbiological basis of endometriosis and chronic endometritis may also support the selective use of antibiotic therapy in cases where infectious endometrial pathology is identified [60].

Endometritis refers to inflammation of the uterine lining and may be infectious or non-infectious in origin. The most common cause is infection, which can extend to the fallopian tubes, ovaries, or pelvic peritoneum, potentially resulting in pelvic inflammatory disease (PID) [62] Acute endometritis is typically associated with sexually transmitted infections (STIs) or bacterial vaginosis (BV) and presents with symptoms resembling PID, including fever, pelvic pain, and abnormal vaginal discharge. Histologically, acute endometritis is characterized by neutrophilic infiltration and microabscess formation within the endometrium [63].

Acute salpingitis frequently accompanies acute endometritis in the setting of PID and is directly associated with tubal infertility due to fibrotic scarring. Chronic endometritis is a recognized contributor to recurrent implantation failure (RIF), recurrent miscarriage (RPL), and infertility. It is often clinically subtle and associated with microbial colonization of the genital tract, plasma cell infiltration of the endometrial stroma, and other features of chronic inflammation [63]. Patients most commonly report abnormal uterine bleeding, dyspareunia, or pelvic pain. Diagnosis is challenging, as histopathological criteria for chronic endometritis are limited, with the presence of plasma cells in the endometrial stroma remaining the primary diagnostic hallmark [63].

Chronic endometrial inflammation has also been associated with non-infectious factors, including intrauterine contraceptive devices, endometrial polyps, and submucosal leiomyomas. Predominant pathogens include *Enterococcus faecalis*, *Escherichia coli*, *Klebsiella pneumoniae*, *Mycoplasma* spp., *Ureaplasma* spp., *Gardnerella vaginalis*, *Pseudomonas aeruginosa*, *Saccharomyces cerevisiae*, and *Candida* spp. [57].

Among sexually transmitted infections that disrupt immune homeostasis in the reproductive tract, human papillomavirus (HPV) is particularly prevalent. HPV infection adversely affects reproductive health by altering local immune responses, inducing inflammation, and causing direct damage to gametes and reproductive tissues. High-risk HPV genotypes may induce DNA damage and oxidative stress in reproductive cells, thereby contributing to infertility and oncogenesis. By compromising the mucosal immune barrier of the genital tract, HPV frequently coexists with other STIs, including *Chlamydia trachomatis* and *Neisseria gonorrhoeae*, further disrupting immune balance [64]. This may contribute to disturbances in reproductive tract immune homeostatis, creating an immunologically hostile environment, promoting inflammation, and increasing susceptibility to pelvic inflammatory disease (PID) [65].

HPV can infect spermatozoa, reducing motility and enabling transfer of viral DNA to the oocyte, which may adversely affect early embryonic development. HPV infection of sperm and transmission to the oocyte and developing blastocyst have been proposed as potential contributors to idiopathic infertility and miscarriage. In women, HPV infection may trigger local immune activation, interfere with embryo implantation, and damage placental trophoblast cells through apoptosis, thereby increasing the risk of miscarriage. HPV is the primary cause of cervical cancer and its precursor lesions; treatment (e.g., cervical conization) may reduce cervical mucus production and impair sperm transport and capacitation. Given these associations, HPV genotyping using liquid-based cytology may be considered in the diagnostic work-up of infertility on an individualized basis [66].

## 5. Lymphocyte Testing and Immunological Tolerance Assays

It should be emphasized that methods often used in scientific research cannot always be directly implemented into routine clinical diagnostics. The net activity of the immune system is dynamic and depends on an immense number of factors, many of which are impossible to account for. Causal inference cannot be based merely on correlations between variables; scientific evidence must be established with certainty and free from paralogisms. Furthermore, before therapeutic methods are introduced into clinical practice, their safety must be confirmed and independently validated. Until diagnostic and therapeutic procedures are thoroughly verified, the appropriate place for these tests remains within clinical research.

### 5.1. Lymphocyte Subpopulations Testing

The assessment of individual lymphocyte populations by flow cytometry enables determination of the immunophenotype, i.e., the concentration and proportions of distinct lymphoid cell lineages in a collected blood sample. This makes it possible to estimate whether the immune system is hyperreactive or whether immunodeficiencies are present. Among women with recurrent miscarriages, disturbances in the number and proportions of specific lymphocyte subpopulations are observed. The recruitment and activity of immunological factors within the reproductive tract of both women and men, and their influence on reproductive processes, depend on the state of the peripheral immune system. In immunophenotyping, the number and percentage of T lymphocytes (CD3+), helper T cells (CD4+), suppressor/cytotoxic T cells (CD8+), B lymphocytes, and NK (CD3–CD56+/16+) are assessed, along with the CD4/CD8 ratio. Shifts in the proportions of these lymphocyte populations may be associated with miscarriages and impaired embryo implantation. The determination of the percentage of NK with strong CD56 expression (CD56^bright^) and NK with weak CD56 expression (CD56^dim^) is particularly important. Both the peripheral NK cell population and the CD4/CD8 ratio, as well as the presence of the HLA-DQA1*05 allele, exert a significant impact on implantation and the course of pregnancy. These parameters are frequently elevated in women with recurrent miscarriages and failed embryo transfers in in vitro fertilization procedures [67].

### 5.2. Th1/Th2 Cytokine Profile

Cytokines are substances produced by specific types of immune system cells. Released into the circulation and tissues, they transmit intercellular signals and exert effector functions depending on the specificity of their receptors. Cytokines and chemokines play a fundamental role during the normal menstrual cycle, influencing the proper function of the ovary and endometrium. They are secreted in granulosa cells by leukocytes that either normally reside in or periodically migrate into ovarian tissue. It has been demonstrated that in women with infertility there is a disruption of the leukocyte balance in the theca layer of ovarian follicles, leading to a cytokine profile distinct from that observed in leukocytes of healthy women [68].

Evaluation of Th1, Th2 cytokine profiles and the Th1/Th2 ratio is useful in diagnosing reproductive disorders at various stages, ranging from the condition of gonadal tissue and gametogenesis to implantation, placental development, and fetal growth. Both pro- and anti-inflammatory cytokines can be assessed, including: IFN-γ, TNF-α, IL-2, IL-4, IL-5, and IL-10. Pro-inflammatory Th1 lymphocytes predominantly produce IFN-γ, whereas Th17 lymphocytes predominantly produce IL-17. These cytokines contribute to the inflammatory milieu that support implantation. Once its role has been fulfilled, this inflammatory state is subsequently attenuated by Th2 and Treg cells, which produce anti-inflammatory cytokines such as IL-4, IL-5, IL-10, and IL-13 [69]. A well-balanced Th2 response is crucial for creating an environment conducive to the continuation of pregnancy, while the modulatory activity of Treg cells protects embryonic and fetal tissues from maternal immune attack. Understanding these processes substantially facilitates interpretation of the obtained results. It should be emphasized that the paradigm of balance along a simple Th1–Th2 axis is a considerable oversimplification derived from concepts formulated 40 years ago. In reality, Th lymphocytes are highly plastic and may respond in divergent ways depending on the context. The nature of the immune response is in practice composed of multiple components, regulated by numerous factors including the microbiome, and is better represented in a multidimensional framework rather than along a single axis [12].

### 5.3. Natural Killer Cells (NK)

Natural killer (NK) cells, classified as innate lymphoid cells, constitute an important population of the immune system with intrinsic cytotoxicity. They participate in the early phases of innate antimicrobial and antitumor responses, as well as in immune surveillance. NK are activated upon contact with cells that lack class I MHC proteins on their surface, or in which their expression is downregulated, as occurs in viral infections or in tumor cells [70]. Systemic NK are cytotoxic, whereas after entering the endometrium they differentiate into uterine NK (uNK) cells and lose their killing activity. They are most abundant in the uterine cavity during the implantation window, constituting approximately 70% of immune cells present [71]. Within the endometrium, two NK fractions exist: strongly cytotoxic NK CD56^dim^ cells and weakly cytotoxic NK CD56^bright^ cells. The local balance of uNK phenotypes plays a pivotal role in maintaining endometrial homeostasis and maternal–fetal immune tolerance. Due to such strong compartmentalization of lymphocytes, the assessment of their peripheral blood concentrations does not provide insight into the uterine microenvironment. In the uterus, uNK secrete cytokines and growth factors that support adhesion and invasion of the embryonic trophoblast into the decidua. They also produce matrix metalloproteinases and angiogenic factors, including VEGF, which regulate the remodeling of uterine spiral arteries and placental formation. Abnormal function, activity, or phenotypic balance of uNK leads to disturbances in the development, structure, and function of the placenta—an essential, temporary organ for the embryo and later the fetus [71].

The quantification and functional assessment of NK—both in peripheral blood by flow cytometry and in the uterus by immunohistochemical analysis of endometrial biopsy specimens—may be considered as part of the diagnostic work-up in selected patients, particularly in the context of implantation failure, fetal growth restriction, preterm birth, and miscarriage [67]. However, causal inference must be approached with caution, and any proposed therapeutic interventions require independent validation before implementation in routine clinical practice. Until diagnostic and therapeutic procedures are thoroughly verified, the most appropriate setting for these tests remains clinical research.

In a retrospective study evaluating the medical records of women with miscarriage, the percentage of peripheral NK and the CD4/CD8 ratio were reported to be elevated in women with recurrent miscarriage and in vitro fertilization—embryo transfer (IVF-ET) failures. Although these findings may suggest immune hyperreactivity, they do not identify the underlying causes of the observed immune phenotype or clarify the source of the presumed autoreactivity reflected by an increased CD4/CD8 ratio and higher peripheral NK cell percentages [67].

Accordingly, any clinical interpretation should be embedded within a comprehensive diagnostic process aimed at identifying systemic drivers of immune dysregulation, including chronic infection and autoimmunity, in line with good clinical practice. The current ESHRE (European Society of Human Reproduction and Embryology) position (2025), consistent with previous guidance, does not recommend routine NK cell testing, largely because peripheral blood measurements do not reflect the uterine immune microenvironment and may prompt unnecessary treatment. Nevertheless, abnormal peripheral lymphocyte profiles (including NK, CD4, and XD8 subsets) may serve as a signal to investigate underlying systemic inflammatory, infectious, or autoimmune conditions, which—when treated causally—may improve fertility potential. As discussed earlier, both autoimmunity and infections can impair oocyte quality and endometrial receptivity. Similarly, local infection or colonization of the reproductive tract may be associated with altered immune cell distributions within the uterus. Therefore, immunomodulatory treatment may be unjustified if based solely on abnormal NK, uNK or other lymphocyte parameters in the absence of clinical evidence identifying the cause of immune dysregulation. This approach may improve the quality of care for infertile couples by supporting more targeted, clinically justified interventions.

### 5.4. Natural Killer Receptors Genotyping—KIR/HLA-C

Uterine NK, which interact with extravillous trophoblast (EVT) cells, play a fundamental role in the development of maternal immune tolerance towards the embryo and fetus. Maternal uNK recognize embryonic human leukocyte antigens (HLA) via their killer-cell immunoglobulin-like receptors (KIR). The best-characterized interactions are those between KIR and HLA-C, which determine pregnancy success. The embryo inherits HLA alleles from both parents; hence paternal genes have a direct and significant impact on uNK activation. Diagnostic evaluation should therefore include both KIR and HLA-C typing in the mother and HLA-C typing in the prospective father. Both molecules—KIR and HLA-C—are highly polymorphic, giving rise to numerous possible maternal–embryonic KIR/HLA-C combinations [72]. This high diversity complicates diagnostics and makes definitive determination of clinical outcomes difficult. Some authors have suggested that individual KIR genotypes, as well as combinations of maternal KIR with embryonic or paternal HLA-C, may exert either beneficial or detrimental effects on pregnancy outcomes [72].

KIR are divided into activating and inhibitory types. Inhibitory KIRs recognize the embryo as an antigenically semi-foreign graft, whereas activating KIRs are indispensable for placental development. Disruption of the balance between inhibitory and activating KIRs may result in complications with pregnancy maintenance. Genes encoding KIR, located on chromosome 19, exhibit high polymorphism. Therefore, to provide definitive evidence of the clinical significance of these associations, studies would need to be conducted on much larger patient cohorts than those performed to date. Three KIR genotypes have been distinguished: AA, AB, BB. The AA genotype contains genes encoding inhibitory receptors, whereas AB and BB genotypes encode both activating and inhibitory receptors. It is postulated that under physiological conditions, inhibitory uNK-KIRs bind to their corresponding HLA-C ligands and transmit signals that suppress uNK cytotoxicity. This creates a maternal immune-tolerant environment essential for fetal survival and development. However, different KIR–HLA-C combinations modulate implantation and subsequent pregnancy progression in distinct ways. HLA-C1 allotypes bind to the inhibitory receptors KIR2DL2 (haplotype B) and KIR2DL3 (haplotype A). By contrast, HLA-C2 allotypes interact with the activating receptor KIR2DS1 (haplotype B) and the inhibitory receptor KIR2DL1 (haplotype A). The most inhibitory maternal genotype—KIR AA—when combined with paternally inherited fetal HLA-C2, excessively suppresses uNK activity, which is associated with an increased risk of pregnancy complications. Paternal HLA-C2 expressed on trophoblasts interacts predominantly with the inhibitory receptor KIR2DL1 on maternal uNK, leading to excessive inhibition of uNK activity. This results in reduced trophoblast invasion of the uterine spiral arteries and abnormal placental development, which may underlie implantation failure (RIF), miscarriage, preeclampsia, or fetal growth restriction. The most unfavorable reaction occurs between KIR AA and HLA-C2C2. The HLA-C1 and HLA-C2 epitopes are recognized by inhibitory receptors such as KIR2DL*1* (for C2) and KIR2DL2/3 (for C1). Therefore, in the case of the KIR AA genotype combined with HLA-C1C2, there is also an increased, albeit lower, risk of pregnancy complications. Conversely, a homozygous HLA-C1C1 fetus in combination with maternal KIR AA does not confer an increased risk of miscarriage or pregnancy complications, as the interaction between HLA-C1 and the inhibitory receptor KIR2DL2/3 is not as strong. The inhibitory signal of uNK is weaker in this context; therefore, arterial development proceeds normally, and trophoblast invasion is appropriate [63].

On the other hand, an activating reaction occurs between uNK-KIR-B and HLA-C1. Activating KIRs, such as KIR2DS1, can also bind HLA-C2, which leads to NK cell activation and may contribute to pregnancy complications at any stage. Thus, both excessive inhibition and overstimulation of uNK activity may threaten normal placental development and subsequent stages of pregnancy. A subtle balance between inhibition and activation of uNK is therefore crucial for successful reproduction.

Nevertheless, the identified KIR–HLA relationships are not clinically straightforward. Genotyping KIR in the female patient alone, without analyzing the partner’s HLA-C status, is not clinically useful in infertility treatment. Furthermore, it must be emphasized that nearly all antigens belonging to the major histocompatibility complex (MHC)—including HLA-G, HLA-DR, and HLA-DQ—are expressed at the maternal–fetal interface, and their polymorphisms carry immunological implications important for successful implantation, placental development, and fetal growth [69]. For example, embryonic HLA-G and maternal HLA-DQ represent important factors in determining the risk of pregnancy complications. The non-classical class I HLA-G molecule, expressed in extravillous trophoblasts (EVT) at the maternal–fetal interface, interacts with uNK. It plays a pivotal role in remodeling of the spiral uterine arteries, placental development, and the establishment of a tolerogenic immunological environment essential for pregnancy [62]. It must be stressed that, as in all comparable cases, drawing phenotypic implications from frequent genotypes is methodologically questionable, and the available evidence for clinical utility is so weak that currently no scientific society recommends such diagnostic testing in routine clinical practice.

However, a higher frequency of *HLA-DQ2/DQ8* polymorphisms has been reported among patients with recurrent pregnancy loss. The presence of this genotype is associated not only with an increased lifetime risk of coeliac disease but also with unfavorable reproductive autoimmune markers such as anticardiolipin antibodies and anti-thyroid peroxidase antibodies [62]. There is, however, insufficient evidence to associate adherence to a gluten-free diet in carriers of this polymorphism with any specific clinical outcome.

Ongoing research in reproductive immunology continues to investigate NK-related genes (members of the leukocyte immunoglobulin-like receptor [LILR] family) and their potential diagnostic utility in obstetric complications, including failed embryo transfers (ET) [62].

The activity of uNK, which is crucial for trophoblast invasion and placental development, is regulated by Treg lymphocytes. These cells play a dominant role in all processes of human reproduction, and their number and functionality depend on the immunomodulatory effects of the pregnancy hormone progesterone.

### 5.5. Cross-Match and Allo-MLR Tests

The cross-match test, also referred to as the microlymphocytotoxicity assay, is sometimes performed in the diagnostic work-up of infertility in cases of conception difficulties, recurrent miscarriages, or prior to in vitro fertilization-embryo transfer (IVF-ET). The test requires cross-reaction of the male partner’s lymphocytes with the female partner’s serum to evaluate the presence of IgG antibodies directed against her partner’s CD3+ lymphocytes. It is believed that such antibodies may similarly react with spermatozoa and the embryo. It has been suggested that the cross-match test is useful in the management of recurrent miscarriage and in assessing the effectiveness of immunotherapy in couples already undergoing treatment for immunological infertility [69]. The cross-match assay—targeting antibodies specific for HLA—should be performed in combination with the evaluation of blocking antibodies (allo-MLR).

Allo-MLR (Mixed Lymphocyte Reaction Test) The Allo-MLR (Mixed Lymphocyte Reaction Test) is used to assess the proliferative response of a woman’s lymphocytes to the lymphocytes of her prospective partner. Under physiological conditions, blocking antibodies appear in the serum of pregnant women between the fourth and twelfth week of gestation, reaching peak concentrations by the end of the second trimester. This test enables the detection of blocking antibodies in maternal serum, which play a crucial role in protecting the developing embryo from the maternal immune system. Those antibodies are expected to reduce the expression of IL-2 receptors in the mother and inhibit T-cell activation, thereby believed to reduce the risk of pregnancy loss of immunological origin. The test result is expressed as the percentage inhibition of the cytotoxic effect in the co-culture of lymphocytes from both partners. Insufficient levels or absence of these antibodies may result in miscarriage, as the maternal immune system may recognize fetal cells as foreign. The mixed lymphocyte reaction test is also intended to monitor the effectiveness of immunization therapy with allogeneic partner lymphocytes [69]. It should be noted, however, that at present such diagnostics and subsequent effectiveness of immunization treatment of the mother’s immune system remain highly controversial and are not recommended for clinical practice by any medical or scientific society.

### 5.6. Immunohistochemical Assessment of CD3, CD4, CD16, CD25, CD56, and CD138 Antigens in Endometrium

Endometrial assessment by immunohistochemistry is habitually performed in the mid-luteal phase to detect immunological abnormalities that may underlie infertility, particularly implantation disorders or early post-implantation loses. Conversely, it should be emphasized that most frequent disorder-subclinical chronic endometritis may be more reliably and conveniently diagnosed in the follicular phase of the cycle [73]. The collected endometrial tissue sample is specially processed for immunohistochemical studies, in which immune cell CD (Cluster of Differentiation) markers are identified and quantitated. The following cell populations can be distinguished: CD3+—T lymphocytes, CD25+—regulatory T lymphocytes, CD56+—NK, CD138+—plasma cells [14].

T lymphocytes (CD3+) are involved in cell-mediated immune responses. Elevated numbers within the endometrium may indicate excessive immunological activity and auto-aggression. This can lead to impaired sperm and embryo transport, as well as abnormalities in implantation and placental formation.

Regulatory T cells (Treg, CD25+) play a central role in all stages of reproduction—from ovulation, fertilization, implantation, pregnancy, through to delivery. They are essential for reproductive success, as they mediate the controlled suppression of inflammation required for blastocyst invasion. Their deficiency or dysfunction contributes to the development of autoimmune responses, late obstetric complications such as preeclampsia and eclampsia, and restricted fetal growth.

Uterine NK (uNK, CD56+) are the most abundant immune cell population in the endometrium. They exert a regulatory function, supplying growth and angiogenic factors crucial for appropriate vascular spiralization and proper placental development. Their significance has been discussed above.

Abnormal numbers or imbalances in the ratio of uNK CD56+ cells to Treg CD25+ cells may indicate the need for immunomodulatory therapy. In contrast, the presence of CD138+ plasma cells is a hallmark of chronic endometritis, reflecting the immune system’s response to infection, and necessitates targeted antimicrobial and anti-inflammatory treatment. Importantly, in clinical practice it is usually assumed that the identification of more than five CD138+ cells per 10 mm^2^ (threshold is much lower than 1 cell in mm^2^ or 1 cell in High-Power Field-HPF) is sufficient to confirm chronic endometritis. In case of chronic endometritis additional CD marker testing becomes unnecessary and may be misleading [74,75].

This diagnostic approach may be particularly useful for women experiencing recurrent miscarriage or repeated implantation failure (RIF). The identification and quantification of specific lymphocyte subpopulations in the endometrium may enable targeted therapeutic interventions.

For some of the tests described in Section 4 and Section 5, the current evidence is weak or contradictory; therefore, major scientific societies do not recommend their use outside clinical research or controlled settings.

## 6. Immunological Diagnostic Evaluation of Male Infertility

It is estimated that up to 50% of infertility cases originate from male factors. Seminal plasma and spermatozoa contain substances with antigenic properties, against which the immune system can generate specific antibodies. These include a variety of proteins, blood group antigens, HLA, lactoferrin present in seminal fluid, and sperm-specific antigens such as hyaluronidase, acrosin, protamine, and LDH-X. A sperm lipoglycoprotein represents one antigen capable of inducing sperm agglutination and immobilization. It is know from decades, that spermatozoa are also strong absorbers of antigens, which further increases the potential for immunization and the production of additional specific antibodies [76]. The male immune system does not develop self-tolerance to semen antigens, as maturation of the reproductive system and spermatogenesis begins during puberty, whereas tolerance to self-antigens is established during early life. The testes are considered an immune-privileged organ, owing to the blood–testis barrier formed by tight junctions between Sertoli cells and between epididymal cells. However, capillaries are not entirely impermeable, and complete immunological isolation is not achieved, despite markedly reduced lymphocyte and antibody migration into the seminiferous tubules. Thus, local immunoregulation within the testes is essential. Sertoli cells suppress activated T lymphocytes and degrade abnormal spermatozoa without presenting their antigens [5]. Disruption of these mechanisms and loss of local immune balance results in increased production of pro-inflammatory cytokines, reduced tolerogenic dendritic cell activity, and T-cell activation, all of which underlie the formation of antisperm antibodies (ASA). Clinical conditions such as varicocele, functional testicular hyperthermia, orchitis, prostatitis, history of cryptorchidism, trauma, and surgical interventions may damage the blood–testis barrier, leading to immune system activation, antibody production, and immunological infertility [21].

Autoimmune orchitis may be: primary, with ASA present in the absence of systemic autoimmune disease, or secondary, developing on the basis of testicular vasculitis associated with systemic autoimmune disorders. Autoimmune orchitis is most often asymptomatic and, similar to women, is frequently classified in practice as idiopathic infertility [77]. Diagnosis typically required testicular biopsy, a procedure no longer commonly used in clinical practice. Alternatively, the identification of anti-testicular antibodies produced during chronic orchitis may be explored [78]. These could potentially replace biopsy, much like thyroid autoantibodies have already replaced biopsy in the diagnosis of Hashimoto’s disease.

The MAR test (Mixed Antiglobulin Reaction, IgG/IgA) is used to detect antisperm antibodies in semen. This test does not need to be routinely performed in every infertile man but is indicated in cases of sperm agglutination or a history of testicular trauma or inguinal surgery. A positive test result alone is insufficient to confirm immunological infertility; evidence of impaired sperm function must also be demonstrated. ASA can be detected in 16% of infertile men and in about 2% of fertile men. They may also be present in serum and reproductive tract secretions of women immunized by semen exposure. However, due to their relatively high prevalence in healthy fertile individuals, the precise clinical significance of ASA remains difficult to establish [21].

The presence of ASA IgA and IgG can reduce the fertilizing ability of spermatozoa by impairing their migration through cervical mucus, causing agglutination, and promoting excessive phagocytosis. This leads to decreased sperm viability and motility and increased agglutination, which can be detected in routine semen analysis. Antisperm antibodies also interfere with spermatogenesis, disrupt the acrosomal reaction, block sperm–oocyte interactions, and impair early embryonic development. Binding of ≥50% of motile spermatozoa with antibody-coated particles is considered a positive test [79,80].

The most common cause of autoimmune orchitis is primary infection. Orchitis usually develops as a result of a local or systemic infection. Even when appropriate antimicrobial and microbiological treatment is administered in the early stages, it may not halt persistent immunological processes and pro-inflammatory cytokine production that damage testicular structures. In particular, viral infections may disrupt spermatogenesis, although confirming a direct causal relationship is difficult because of heterogeneous infection patterns. Viral orchitis is much rarer than secondary orchitis, which is often caused by ascending bacterial infections of the genitourinary tract [81].

Disruption of the testicular immune barrier may facilitate microbial transmission and infection. Antibodies and biofilm in the reproductive tract epithelia can damage germ cells and impair spermatogenesis and sperm maturation [82]. Transmission of bacteria is relatively easy through the common passageway of the urethra used for both urine excretion and semen ejaculation. The prostate contributes ~90% of seminal fluid volume. Not only overt infections, but also microbial carriage within the prostate can trigger immune cell influx and local release of inflammatory mediators, cytokines, and antibodies [81]. Chronic prostatitis leads to increased IL-6 secretion, which induces oxidative stress, causing sperm damage and lowering semen quality [83].

An abnormal microbiome in external cavities such as the reproductive tract, oral cavity, and gastrointestinal tract, as well as sexually transmitted diseases (STDs) and common Gram-positive bacilli in the urogenital area of patients and their partners, may trigger local immune activation and sperm damage [39].

Viral infections are particularly conducive to immunological infertility. Many viruses in semen, such as HHV-6, may remain asymptomatic or latent without impairing reproductive function. Some viruses, like influenza virus, are not localized in the male reproductive system but can still impair fertility. Influenza infection reduces sperm count and motility and alters morphology through apoptosis and direct sperm DNA damage, as well as by activating immune cells, increasing pro-inflammatory cytokines, and inducing fever, which can suppress fertility for several months [84]. Clinically, mixed and complex viral infections are much more frequently observed. The presence of viruses also predisposes to easier co-infection with other pathogens due to weakened local defense mechanisms [84].

Certain viruses with strong tropism for the male reproductive system may be sexually transmitted, such as SARS-CoV-2. This virus infects organs through ACE2 receptors and enters Leydig and Sertoli cells. It causes extensive cell destruction, thickening of basement membranes, and leukocyte infiltration in the seminiferous epithelium, impairing spermatogenesis. Interstitial increases in pro-inflammatory cytokines (IL-6, TNF-α, MCP-1) lead to their release in semen of COVID-19 patients, reducing sperm concentration and motility. The local immune response induced by the virus may trigger autoimmunity, leading to autoimmune orchitis that damages the spermatogenic epithelium. The accumulation of inflammatory cells and cytokines, along with fever, may damage germ cells, lower testosterone levels, and impair fertility in COVID-19 patients [85].

Other viruses, such as mumps virus, trigger signaling via RIG-I (retinoic acid-inducible gene I-like receptor) and TLR2 (Toll-like receptor 2), activating the antiviral response through type I interferon (IFN-I) and inflammatory cytokine production. Mumps infection can induce the production of antiosperm antibodies and have long-term adverse consequences for fertility [86].

All viruses may impact semen quality and fertility through multiple pathways, including: direct effects on spermatozoa or germ cells, disruption of testicular function, induction of reproductive tract inflammation, and the triggering of immune responses.

Similarly, STDs such as Chlamydia trachomatis and Neisseria gonorrhoeae are common pathogens causing orchitis. During infection, DNA and transcriptional damage occur in germ cells, alongside epigenetic alterations that disrupt the sperm epigenome. Increased leukocyte infiltration damages the blood–testis barrier, reduces sperm count and seminiferous tubule volume, leading to low fertility and fetal abnormalities [87].

Semen of patients with varicocele often shows colonization by *Ureaplasma urealyticum*, *Chlamydia trachomatis*, *Mycoplasma hominis*, *Toxoplasma gondii* and a higher prevalence of viruses: HSV, CMV, HBV and HCV. The microorganisms themselves can damage reproductive structures and elements but, most importantly, the oxidative stress caused by their presence and the influx of inflammatory mediators may have an adverse effect on semen quality [82]. Patients with varicocele have significantly lower sperm concentration and total sperm count than healthy individuals, as well as lower chromatin condensation, higher DNA fragmentation, and a reduced percentage of sperm with low mitochondrial membrane potential (MMP). This is consistent with changes observed in leukocyte subpopulations in semen of patients with varicocele, which lead to increased production of pro-inflammatory cytokines in semen [88].

In the diagnosis of male immunological infertility, autoimmune diseases must also be considered, although they are generally less frequent than in women. In this patient group, higher levels of gonadotropins and varicocele were observed more often, along with abnormal semen parameters with sperm DNA fragmentation [80]. Autoimmune diseases and the presence of antibodies should be diagnosed in the same way as in women to enable the implementation of appropriate therapy. On the other hand, immunomodulatory drugs used to treat the disease should be selected in such a way as not to negatively affect spermatogenesis and reproduction [21].

## 7. Conclusions

Infertility is not a uniform disease but a multifactorial disorder. Abnormalities in the menstrual cycle and reproductive system functioning stem not only, as previously assumed, from defects of the reproductive organs or hormones, but also from immunological disorders. The key stages of reproduction are initiated by local inflammation, regulated by immune cells. Dynamic and properly modulated immune responses, mediated by progesterone-dependent regulatory T cells, ensure the resolution of local inflammation and the correct continuation of reproductive processes. In the etiology of infertility, low-grade systemic inflammation must be considered, arising in the course of systemic immune responses associated with infections, metabolic disorders, insulin resistance, endometriosis, and oxidative stress. Main causes and mechanisms of those disturbances were briefly summarized in Table 1.

It appears that obstetric failures may result from an excessively strong maternal immune response to the developing embryo/fetus as a foreign organism.

It should be remembered that many immunological disturbances hindering fertilization, implantation, and pregnancy can be eliminated by improving the general condition of the body, hormonal and metabolic balance, removing inflammation, treating autoimmunity, and correcting deficiencies of vitamins and microelements. Alongside pharmacological intervention, these measures should be supported by changes in diet and lifestyle in infertile individuals. Currently, it is believed that interaction with the gut microbiota plays a crucial role in the precise regulation of the immune system and in the development of many inflammatory diseases. The influence of microbiota and the possible therapeutic opportunities related to it are extremely interesting and may revolutionize the treatment of infertility in the future. However, these issues go far beyond the scope of this article.

It is also important not to forget psychological factors and the necessity of regular intercourse in couples trying to conceive, with a frequency of 2–3 times a week, to ensure proper spermatogenesis, sperm maturation, and maternal tolerance to paternal antigens. Many diagnostic aspects still require further validation, and therapeutic methods must be independently verified before being introduced into routine clinical practice. Further research will make it possible for in-depth, multi-dimensional diagnostics, considering the crucial role of immunological factors in procreation, to improve treatment outcomes and reduce the suffering of couples affected by infertility.

A graded diagnostic approach is proposed, emphasizing that assessment of complex immunological processes such as cytokine profiling or immune signal transduction should not precede the exclusion of basic systemic disorders and infectious causes. Some immunological tests have an established role in infertility diagnostics, whereas others should be reserved for carefully selected cases in which measurable clinical benefit is anticipated. Certain tests remain investigational and are not recommended for routine practice pending further validation.

Detection and correction of metabolic and hormonal disturbances or eradication of infectious agents will always be reflected in an improvement of immune system function. Importantly, immune disturbances affecting reproduction are often secondary and may be resolved through treatment of underlying non-immunological conditions. In most cases, there is no necessity to employ complex immunological tests that are difficult to access or interpret.

This review therefore outlines general and infectious conditions that complicate reproductive immunology, the explanation and treatment of which may yield measurable clinical benefits and restore immune balance. The purpose of this publication is to provide a framework for justifying immunological testing in infertility, rather than advocate universal immunological screening.

## Figures and Tables

**Table 1:** Immunological causes of infertility, potential diagnostic tests and relevance levels of the outcome were shown in the table.

Diagnostic Area	Immunological Mechanism	Diagnostic Methods	Clinical Relevance	Ref. No.
**Hormonal Regulation**	Progesterone and estrogen modulate Th1/Th2 and Treg balance	Hormone level assessment, luteal phase evaluation	Progesterone deficiency → implantation failure, miscarriage	[1,4,17,18,19]
**Autoimmune Diseases**	Autoantibodies (ANA, anti-TPO, anti-TG, APS antibodies, anti-hCG, anti-FSH)	ANA panel, antiphospholipid antibodies, thyroid antibodies	Disturbed oocyte/embryo development, implantation disorders, miscarriage	[20,27,30,40]
**Inflammation/Infection**	Chronic or subclinical infection; microbial dysbiosis (Ureaplasma, Chlamydia, Mycoplasma)	PCR, cultures, immunohistochemistry, cytokine profiling (IL-6, IL-1β)	Inflammatory cytokines disrupt implantation, reduce oocyte/sperm quality	[6,7,57]
**Endometriosis and PCOS**	Chronic inflammation, progesterone resistance, autoantibodies	Cytokine and immune profile evaluation, laparoscopic diagnosis	Infertility caused by immune and endocrine disorders	[1,2,41]
**Lymphocyte** **Subpopulations**	Abnormal T-cell, B-cell, NK-cell ratios (CD3+, CD4+, CD8+, CD56+)	Flow cytometry (immunophenotyping), CD4/CD8 ratio	Related to recurrent miscarriages, implantation failure	[67]
**Cytokine Profile (Th1/Th2)**	Imbalance between Th1 (pro-inflammatory) and Th2/Treg (anti-inflammatory) cytokines	Cytokine panel: IFN-γ, TNF, IL-2, IL-4, IL-5, IL-10	Th1/Th17 hyperactivity → implantation failure; Th2/Treg dominance → pregnancy maintenance	[69]
**NK and KIR/HLA-C**	Disturbances in NK cell phenotype (CD56^bright/dim^) or KIR/HLA-C incompatibility	Flow cytometry, molecular KIR/HLA-C typing	Excess inhibition or activation → RIF, miscarriage, preeclampsia	[62,71,72]
**Cross-Match and Allo-MLR**	Absence of blocking antibodies against paternal antigens	Cross-match assay, Mixed Lymphocyte Reaction (MLR)	Linked to recurrent miscarriage, embryo rejection	[69]
**Endometrial IHC**	Abnormal immune cell populations in endometrium	Biopsy + CD marker panel: CD3, CD25, CD56, CD138	Chronic endometritis, immune imbalance, implantation defects	[74,75]
**Male Immune Infertility**	Antisperm antibodies (ASA), autoimmune orchitis, infections	MAR test (IgG/IgA), semen analysis, antibody detection	Reduced motility, sperm agglutination, impaired fertilization	[5,21,77]
**Systemic Factors**	Oxidative stress, metabolic inflammation, obesity, insulin resistance	ROS assays, metabolic profile, vitamin/micronutrient panels	Disruption of Treg function, poor oocyte/sperm quality	[22,28]
**Microbiome and Gut–Reproductive Axis**	Dysbiosis affects immune modulation	Microbiome sequencing, stool analysis	Impaired hormonal and immune balance → infertility	[39]

## Data Availability

No new data were created or analyzed in this study.

## Abbreviations

The following abbreviations are used in this manuscript:

GUMED	Medical University of Gdańsk
CMKP	Centre of Postgraduate Medical Education
Th	T helper lymphocyte
NK	Natural Killer Cells
WHO	World Health Organization
DOI	Digital Object Identifier
RIF	recurrent implantation failure
RPL	recurrent pregnancy loss
Treg	regulatory T cells
ANA	Antinuclear Antibody
ASA	Antisperm Antibody
APS	Antiphospholipid Syndrome
MMP	Mitochondrial Membrane Potential
LUF	luteinized unruptured follicle
IL	Interleukin
hCG	Human Chorionic Gonadotropin
MLR	Mixed Lymphocyte Reaction
HLA	Human Leukocyte Antigens
KIR	Killer-cell Immunoglobulin-like Receptors
ROS	Reactive Oxygen Species
DC	Dendritic Cells
LH	Luteinizing Hormone
CD	Clusters of Differentiation
RIG-I	retinoic acid-inducible gene I-like receptor
TLR	Toll-like receptor
STD	Sexually Transmitted Disease
MAR	Mixed Antiglobulin Reaction
HPF	High-power Field
LILR	leukocyte immunoglobulin-like receptor
EVT	extravillous trophoblast
ET	Embryo Transfer
IVF	In-vitro fertilization
POI	Premature Ovarian Insufficiency
anti-TPO	anti-thyroid peroxidase antibodies
anti-TG	anti-thyroglobulin antibodies
TRAb	anti-thyrotropin receptor antibodies
NAWA	National Agency for Academic Exchange
MAGI	Male Accessory Gland Infection
PCOS	Polycystic Ovary Syndrome
MHC	Major Histocompatibility Complex
IFN	Interferon
